# Physicians’ satisfaction with clinical referral laboratories in Rwanda

**DOI:** 10.1080/16549716.2020.1834965

**Published:** 2020-11-20

**Authors:** Vincent Rusanganwa, Jean Bosco Gahutu, Anna-Karin Hurtig, Magnus Evander

**Affiliations:** a College of Medicine and Health Sciences, University of Rwanda, Kigali, Rwanda; b Department of Clinical Microbiology, Virology, Umeå University, Umeå, Sweden; c Ministry of Health, Department of Planning, Health Financing and information System, Kigali, Rwanda; d Department of Epidemiology and Global Health, Umeå University, Umeå, Sweden

**Keywords:** Laboratory services, physician satisfaction, Rwanda, health system, quality healthcare

## Abstract

**Background:**

The quality of laboratory services is crucial for quality of patient care. Clinical services and physicians’ decisions depend largely on laboratory test results for appropriate patients’ management. Therefore, physicians’ satisfaction with laboratory services is a key measurement of the quality service that stresses impactful laboratory service improvement to benefit patients.

**Objective:**

To assess physicians’ satisfaction and perspectives on the quality of services in clinical referral laboratories in Rwanda.

**Methods:**

A cross-sectional survey among physicians from four referral hospitals with closed-ended questionnaire and one general open-ended question. A five-point Likert scale rating was used to measure satisfaction. Descriptive, ordered logistic regression, and thematic analysis were used.

**Results:**

In total, 462 of 507 physicians (91% response rate) participated in the study. Overall mean satisfaction was 3.2 out of 5, and 36.2% of physicians were satisfied (satisfied and strongly satisfied) with laboratory services. In four service categories out of 17, the physicians’ satisfaction was over 50%. The categories were: reliability of results (69.9%), adequacy of test reports (61.9%), laboratory staff availability (58.4%), and laboratory leadership responsiveness (51.3%). Lowest satisfaction was seen for routine test turnaround time (TAT) (19.3%), in-patient stat (urgent) test TAT (27%), communication of changes such as reagent stock out, new test (29%), and missing outpatient results (31%). Eighty-four percent answered that test TAT was not communicated, and 73.4% lacked virology diagnostics. Pediatricians, internists, and more experienced physicians were less satisfied. While ineffective communication, result delays, and service interruption were perceived as dissatisfying patterns, external audits were appreciated for improving laboratory services.

**Conclusion:**

Availing continuously laboratory tests, timely result reporting, and effective communication between laboratories and clinicians would increase physicians’ satisfaction and likely improve the quality of health care. Laboratory staff participation in clinical meetings and ward rounds with physicians may address most of the physicians’ concerns.

## Background

Clinicians are central to patient health care. Ideally, the patient healthcare process is initiated and concluded with a physician [1]. The diagnosis is based on clinical and paraclinical information, such as laboratory test results. This helps the physician to decide on the management of the patient’s condition. This process of patient health care places the physician in the best position to appreciate the service delivered by clinical laboratories.

The interaction and complementarity between physicians and clinical laboratories should be effective to ensure the quality of patients’ health care The quality of services to patients will be improved with effective collaboration between laboratories and clinicians in response to patients’ needs [2]. Effective communication between these services will likely identify gaps in the accuracy and reliability of test results, as well as in reporting and timeliness [3]. Furthermore, joint problem-solving will be enabled to increase safety and patient-centeredness as well as improvement in other healthcare quality domains. Thus, customer satisfaction, including physicians, will be boosted [1,2]. Therefore, regular customer satisfaction assessments are important in the measurement of quality of health care [3].

Customer satisfaction is established as a laboratory medicine requirement for quality and competence [4]. Physicians are the primary customers of laboratory medicine [3]. Therefore, the level of physicians’ satisfaction with different services of clinical laboratories is a good indicator of the quality performance of laboratory services. Such measurements indicate the needs of customers and laboratory areas of improvement for the quality of patients’ health care. In the USA, laboratory customers’ satisfaction surveys, including physicians are conducted regularly as laboratory accrediting organizations set this as a requirement for clinical laboratory accreditation [3,5–7]. However, such assessment, though contributing to the quality of laboratory services, is not emphasized in other parts of the World including in Africa. As many clinical laboratories in Africa are in the process of improving their quality services, they should evaluate the needs expressed by their clients [8]. Customers’ satisfaction assessments, such as physicians’ ones, would link the current status of the laboratory quality improvement with real customers’ expectations.

In Rwanda, the quality of health care is at the heart of health sector and it is recurrent in its planning circle. The accreditation of health facilities has been ongoing for more than a decade [9–14]. Starting in 2010, clinical referral laboratories embarked on the accreditation process and have received different quality performance scores over the years [9–11]. However, physicians’ perspectives on the quality of laboratory services have not yet been documented. This study aimed to assess physicians’ satisfaction and their perspectives on the quality of services in four clinical referral laboratories in the country.

## Methods

### Study setting

The Rwandan healthcare system is organized in referral and counter-referral system. Four national referral hospitals are on the top of the healthcare referral system pyramid. This study was limited to these four hospitals. The laboratories of these hospitals, as well as the National Reference Laboratory (NRL) (not included in this study) are in the category of national referral laboratories in the country. The four laboratories at these hospitals mainly analyze samples of patients referred to these hospitals. The NRL does not receive patients, instead samples from others health facilities for quality control and some specialized tests such as sequencing and viral load, e.g. HIV, hepatitis C and B. The NRL is also responsible for outbreak investigations, disease surveillance and involved in research. The four hospital laboratories are departments, among others, within their hospitals. They are equipped with modern laboratory equipment. Recent studies in these laboratories did not find that personnel and finance were a challenge for quality [11,15]. The four laboratories are technically supervised by the NRL. In return, the referral hospitals have the mandate of supervising lower level hospitals in their catchment areas, including their laboratories. The four hospitals are named hospitals 1 to 4. Hospital 1 has 500 beds, hospital 2 has 509 beds, hospital 3 has 161 beds, and hospital 4 has 335 beds. Their bed occupancy rates for July 2018 to June 2019 were 76, 79, 87, and 80.2%, respectively (hospital data). The affiliated laboratories performed a monthly average volume of 20,917; 43,127; 54,256 and 32,083 tests, respectively, in the same period (hospital data). These tests were performed in different units such as biochemistry, immunology, serology, hematology, parasitology, bacteriology, histopathology, molecular biology, and hormonology. Genetics tests are performed in one laboratory. The tests performed in these laboratories are shown in the Appendix A.

### Study design

A cross-sectional questionnaire survey to assess the level of physicians’ satisfaction with laboratory services in the four referral hospitals was conducted. A five-point Likert-type scale rating was used as follows: strongly dissatisfied (1 point), dissatisfied (2 points), neutral (3 points), satisfied (4 points) and strongly satisfied (5 points).

### Data collection

A structured questionnaire was elaborated based on College of American Pathologists Q-Probe studies [3]. The questionnaire was pre-tested with physicians at a provincial hospital prior to data collection. Based on minor comments from the pre-test, minor comprehension adjustments were made. The survey focused on 28 questions, eight on the participants’ socio-demographic characteristics and 20 on laboratory services. Among the 20 questions, 17 focused on the level of satisfaction and one was open-ended to provide any relevant additional information which could not be captured with closed-ended questions. The question was formulated as follows: “Please include additional comments regarding the above questions and any other comments you have regarding laboratory service at your hospital.’ The remaining two questions were on turnaround time (TAT) communication with ‘yes,’ ‘no,’ and ‘maybe’ response options, and on the most lacking infectious services with the option of selecting one or more services.

Preparatory visits were made to study sites to explain the study’s objectives to the hospital leadership and physicians. The participants included consultants, residents, and general practitioners from the four referral hospitals. All eligible physicians were invited to voluntarily participate in the survey. Medical doctors working in laboratories and other diagnostic units as well as those at hospital leadership positions were not included in the study.

Participants’ e-mail addresses were obtained from the hospitals’ administration for consultants and general practitioners, while for residents they were obtained from their representatives. An online self-administered questionnaire was distributed to 507 eligible physicians from the four hospitals through their individual e-mails. The data collection started from March to July 2019. The follow-up was done after three weeks from initial distribution of questionnaire. The follow-up combined site visits to meet medical doctors at their respective departmental morning meetings, and electronic messages were sent as reminders. Out of 507 physicians, 462 responded and returned the questionnaire, which represented 91% of participation.

### Data analysis

Stata statistical software version 13.1 was used for descriptive statistics and logistic regression analysis. To highlight the main emerging three categories of satisfaction levels, a five-point Likert scale was collapsed into a three-point scale. The numbers of strongly dissatisfied and strongly satisfied ratings were very low, 33 and 87, respectively, out of 7,854 total ratings in 17 laboratory service categories. Therefore, in the three-point scale, strongly dissatisfied and dissatisfied in the Likert five-point scale were combined to the rating dissatisfied. Further, satisfied and strongly satisfied were combined to satisfied. The neutral ratings were not combined with any other ratings and stood on its own in three-point scale as in the five-point scale. For the department variable, some departments were merged due to small numbers of physicians. Psychiatric and ophthalmology were merged to internal medicine, ear nose, and throat were merged into surgery, and the emergency department to anesthesiology. The percentage on laboratory tests turnaround time (TAT) communication was calculated by combining ‘no’ and ‘maybe’ responses for TAT not communicated and ‘yes’ responses for TAT communicated. The proportion of the most lacking infectious laboratory service was calculated based on number of selections of each specific laboratory infectious service (virology, bacteriology, parasitology, and other infectious disease).

Given the three categories of the outcome, an ordered logistic regression was conducted. First, a bivariate regression analysis between the overall satisfaction and the predictor variables was performed. Then, variables with significant associations (p-value <0.05) were introduced into a multivariate regression model. To validate the model, predictor variables were tested for multicollinearity using the variance inflation factor (VIF). No collinearity was present since the VIF was below 2 for all predictor variables. The proportional odds assumption was also tested with a likelihood ratio test and the results were not significant (P = 0.14) indicating that the proportional odds assumption was valid.

With regard to the opened-ended question, 135 physicians answered the question. We analyzed these data through an inductive thematic approach as described by Braun & Clarke [16]. Statements were read several times, meaning units were highlighted, and similar ones were grouped and coded. Similar codes were identified to form subcategories. With back-and-forth reviews of texts, codes, and subcategories, we generated categories. From the categories with the same patterns, the four following themes emerged: 1) improved laboratory services, 2) ineffective communication 3) delayed results, and 4) unavailability of services.

### Ethical considerations

The research proposal was approved by the Rwanda National Ethics Committee with reference N^o^ 0059/RNEC/2017 and 111/RNEC/2018 and the research project was authorized by the Rwandan Ministry of Health with reference N^o^ 20/1346/DGPHIS/2017. Further authorizations were obtained from the respective leadership of the participating hospitals. Moreover, the first author was introduced by hospital leaders to physicians. In addition, participants had the opportunity to discuss and ask questions about the study at different meetings during the site visits. Participation was voluntary and responses were anonymous, so, respondents were not traceable.

## Results

In total, 462 physicians participated in the survey. Consultants represented 35.9% (166), residents represented 59.9% (275), and general practitioners represented 4.6% (21). This number of general practitioners translates their limited employment in these specialized hospitals. The majority of participants were males (76.8%), reflecting the current gender distribution in the profession in the country. Most of the study participants (89.8%) were aged between 25 and 45 years (Table 1).

**Table 1.** Socio-demographic characteristics of participants

Demographic characteristics (n = 462)	Frequencies	Percentage
Institutions		
Hospital-1	92	19.9
Hospital-2	238	51.5
Hospital-3	67	14.5
Hospital-4	65	14.1
Gender:		
Male	355	76.8
Female	107	23.2
Age:		
25–35	272	58.9
36–45	143	31.0
46–55	35	7.6
56–65	12	2.6
Physician categories		
Specialists(consultants)	166	35.9
Residents	275	59.5
General practitioners	21	4.6
Years of experience		
<5	150	32.5
5–15	275	59.5
16–25	24	5.2
26–35	13	2.8

The proportions of physicians’ satisfaction for the 17 laboratory service categories are displayed in Table 2 as well as the mean satisfaction score for each service. The mean satisfaction score for the overall laboratory services was 3.2 out of 5, and of 462 physicians, 167 (36.2%) were satisfied (satisfied and strongly satisfied combined) with these services. The proportion of physicians’ satisfaction (satisfied and strongly satisfied combined) in the 17 laboratory service categories, varied from 19.3% to 69.9%. The highest appreciated service category was quality/reliability of test results where 69.9% (323 of 462) were satisfied, while the least was the routine tests TAT, where only 19.3% (89 of 462) were satisfied. For the adequacy of test result reports, 61.9% (286 of 462); the availability of laboratory staff, 58.4% (270 of 462) and laboratory leadership responsiveness, 51.3% (237 of 462) of physicians were satisfied (satisfied and strongly satisfied combined). In their comments, the participants revealed their appreciation of laboratory improvements brought by laboratory external assessments and the online platform, where laboratory test requests and reporting of results are done. It was also commented on that these laboratories were well equipped. *‘Laboratories are doing a great achievement with current external survey where they scored three stars, but some tests should be included to meet the physicians’ demand.’*

**Table 2.** Aggregate of physicians’satisfaction and mean scores in laboratory services

	Satisfied^f^	Neutral	Dissatisfied^g^	Mean of Likert score
Laboratory service categories (n = 462)	No. (%)	No. (%)	No. (%)	(SD)
Overall satisfaction	167*(36.2)*	208(45.0)	87*(18.8)*	3.2(0.8)
Availability of laboratory tests	151(32.7)	198*(42.9)*	113*(24.5)*	3.1(0.8)
Availability of infections tests	157*(34.0)*	179*(38.7)*	126 *(27.3)*	3.1(0.9)
Lab mgt ability to find solutions^a^	173*(37.5)*	190*(41.1)*	99*(21.4)*	3.2(0.9)
Courtesy of laboratory staff	217*(47.0)*	182*(39.4)*	63*(13.6)*	3.4(0.9)
Availability of laboratory staff	270*(58.4)*	145*(31.4)*	47*(10.2)*	3.6(0.9)
Laboratory leadership responsiveness	237*(51.3)*	160*(34.6)*	65*(14.1)*	3.3(0.9)
Collaboration of laboratory staff	191*(41.3)*	164*(35.5)*	107*(23.2)*	3.2(1.0)
Critical values notification	177*(38.3)*	122*(26.4)*	163*(35.3)*	3.1(1.2)
Communication of changes^b^	134*(29.0)*	156*(33.8*)	172*(37.2)*	2.9(1.1)
Routine tests TAT^c^	89*(19.3)*	197*(42.7)*	175 *(38.0)*	2.7(0.9)
Inpatient stat test TAT^d^	125*(27.1)*	191*(41.3)*	146*(31.6)*	2.9(0.9)
Sensitivity for emergency tests^e^	149*(32.3)*	165*(35.7)*	148*(32.0)*	3.0(1.0)
Reliability of laboratory tests	323(69.9)	121*(26.2)*	18*(3.9)*	3.8(0.8)
Adequacy of laboratory tests reports	286*(61.9)*	135*(29.2)*	41*(8.9)*	3.7(0.8)
Missing laboratory results in outpatient	143*(31.0)*	228*(49.4)*	91*(19.7)*	3.1(0.9)
Missing laboratory results in inpatient	177*(38.3)*	191*(41.3)*	9*(20.4)*	3.2(0.9)

^a^Laboratory management ability to find solutions.

^b^Changes in laboratory that may guide clinicians to adapt their laboratory request.such as reagent stock out, new tests, equipment broken, etc

^c^TAT is Turnaround time.

^d^Stat test is a test that needs an urgent result for decision on management of patient(s).

^e^Laboratory service and staff sensitivity for emergency tests requests.

^f^Satisfied is the combination of satisfied and strongly satisfied from the Likert five-point scale.

^g^Dissatisfied is the combination of dissatisfied and strongly dissatisfied from the Likert five-point scale.

Apart from routine test TAT, the service categories where the lowest proportion of physicians were satisfied (satisfied and strongly satisfied combined) were in-patient stat test TAT (27.1%), communication of changes in laboratories (29%), outpatient missing test results (31%), and availability of laboratory tests (32.7%) (Table 2). When asked whether laboratories communicated tests TAT to them, 84% (388 of 462) of physicians reported that they were not communicated tests TAT, and only 16% (74 of 462) confirmed to have received that communication. This ineffective communication was also a pattern emphasized by almost all respondents for the open-ended question. The communication of TAT and changes in laboratories, such as added tests or the unavailability of tests for different reasons, were reported as a weakness to be addressed. ‘*Our laboratories work in routine way, no clear connection with clinicians and no concerns raised while wrong or suspicious results. No alert for stock out or changes in procedure to prevent misunderstand with clinicians*’.

Almost all physicians who provided comments reported their concerns about delays in receiving the laboratory results. According to the respondents, some results are issued afterward when they no longer inform patient management. The issue of missing samples was also reported and contributed to delays. Others reported that such delays affected especially emergency, intensive care, and outpatient services due to their service nature and organization. ‘*There needs to be less waiting time for all laboratory tests, but mostly for the critically ill patients. If there could be a separate laboratory service for emergency and intensive care unit with immediate reporting of every result, it can be better. The good thing is that patients don’t wait for payments for emergency tests to be done, once they are recorded in the hospital system*’.

Some services were reported to be unavailable mostly due to reagent stock out, but also to inexistence of such a service. Infectious diseases are some of the most common causes of disease in the region. Physicians were asked to point out which diagnostic tests were missing for infectious disease diagnostics. Of the physicians, 73.4% (339 of 462) answered that virology diagnostics were lacking for their clinical activities. The services related to bacteriology, parasitology, and other infectious diseases were reported as lacking by 32.7%, 14.7%, and 33.8% of respondents, respectively. Regarding comments from respondents, the majority underlined running out of reagents, especially microbial cultures for bacteriology and some biochemistry tests, such as electrolytes. The lack of virology tests in laboratory services was highlighted as a concern. ‘*The biggest issue is for mandatory tests that are not being done in referral hospital. It is hard to us attending personnel to explain it to patients; sometimes ourselves don’t understand. Many tests are still missing as well as stock-out that is on and off’. ‘Generally, our laboratory has improved a lot, it should also bring in missing tests like those for virology such as Parvovirus, Epstein Barr virus and others*’.

The association of socio-demographic characteristics and overall satisfaction was tested (Table 3). In the crude model, physicians in certain institutions, female doctors, those with more than 10 years of experience, and specialties like pediatrics and internal medicine reported a lower level of satisfaction (satisfied and strongly satisfied combined) with the laboratory services. In the adjusted model, the odds of being satisfied was statistically higher at hospital-2 (OR = 1.84, 95% CI: 1.11–3.05) compared to the reference hospital (hospital-1). Those physicians with more than 10 years of experience (OR = 0.32, 95% CI: (0.11–0.90) and those from the pediatric department (OR = 0.44, 95% CI: 0.24–0.82) had however lower odds of satisfaction compared to those with less than 1 year and the anesthetic department, respectively. Higher odds of satisfaction were found among general practitioners (OR = 2.73, 95%CI: 0.88–8.46) and foreign doctors (OR = 1.47, 95% CI: 0.65–3.33), although this was not statistically significant.

**Table 3.** Bivariate and multivariate analysis to assess predicator variables for physicians’ overall satisfaction

General satisfaction				Ordered logistic regression	
Demographic variables	Satisfied^a^ No. (%)	Neutral No. (%)	Dissatisfied^b^ No. (%)	Crude OR(CI)*	Adjusted OR(CI)
**Institutions**					
Hospital-1	31(33.7)	43(46.7)	18(19.6)	1	1
Hospital-2	109(45.8)	89(37.4)	40(16.8)	1.53(0.97–2.42)	1.84(1.11–3.05)
Hospital-3	16(23.9)	40(59.7)	11(16.4)	0.82(0.46–1.41)	0.89(0.46–1.71)
Hospital-4	11(16.9)	36(55.4)	18(27.7)	0.52(0.29–0.95)	0.71(0.38–1.34)
**Gender**					
Male	137(38.6)	155(43.7)	63(17.8)	1	1
Female	30(28.0)	53(49.5)	24(22.4)	0.66(0.44–1)	0.91(0.59–1.42)
**Age**					
25–35	101(37.1)	120(44,1)	51(18.8)	1	
36–45	49(34.3)	64(44.8)	30(21.0)	0.87(0.58–1.28)	
46–55	14(40.0)	16(45.7)	5(14.3)	1.19(0.61–2.30)	
56–65	3(25.0)	8(66.7)	1(8.3)	0.90(0.32–2.49)	
**Physician categories**					
Specialists (Consultants)	49(29.5)	89(53.6)	28(16.9)	1	1
Residents	104(37.8)	114(41.5)	57(20.7)	1.15(0.80–1.65)	0.84(0.50–1.42)
General practitioners	14(66.6)	5(23.8)	2(9.5)	3.88(1.50–10)	2.73(0.88–8.46)
**Years of experience**					
<1	44(37.6)	52(44.4)	21(18.0)	1	1
1–5	90(34.0)	126(47.6)	49(18.5)	0.89(0.59–1.33)	0.74(0.47–1.17)
6–10	27(56.3)	12(25.0)	9(18.8)	1.80(0.92–3.5)	1.23(0.55–2.73)
>10	6(18.8)	18(56.3)	8(25.0)	0.51(0.25–1.05)	0.32(0.11–0.90)
**Nationality**					
Rwandan	147(34.4)	196(45.9)	84(19.7)	1	1
Non-Rwandan	20(60.6)	10(30.3)	3(9.1)	2.86(1.40–5.9)	1.47(0.65–3.33)
**Departments**					
Anesthesiology	37(44.6)	35(42.2)	11(13.3)	1	1
Pediatrics	19(22.9)	39(47.0)	25(30.1)	0.35(0.20–0.64)	0.44(0.24–0.82)
Obstetrics & Gynecology	25(39.1)	25(39.1)	14(21.9)	0.69(0.37–1.30)	0.77(0.39–1.51)
Internal medicine	33(29.0)	55(48.3)	26(22.8)	0.50(0.3–0.87)	0.64(0.36–1.14)
Surgery	50(43.9)	53(46.5)	11(9.7)	1.04(0.61–1.79)	1.23(0.69–2.19)

*OR: Odds Ratio.

CI: Confidence Interval.

^a^Satisfied is the combination of satisfied and strongly satisfied from the Likert five-point scale.

^b^Dissatisfied is the combination of dissatisfied and strongly dissatisfied from the Likert five-point scale.

## Discussion

This study showed that, in four referral hospitals in Rwanda, 36.2% (167 of 462) of physicians were generally satisfied with laboratory services while 18.8% (87/462) were dissatisfied and 45% (208/462) were neutral. Considering the Likert scale mean score for overall satisfaction (3.2 out of 5) (Table 2), this was not far from what reported from similar studies in Ethiopia (3.36; 3.58) and in Alexandria in Egypt (3.46) as well as in Yemen (3.30) [17–20]. The contexts, sample sizes, and different categories of health providers surveyed in some of these studies may explain the small differences in mean scores. However, this mean score was less than those reported in USA (4.1 and 4.4) [3,6].

The findings of the survey indicated that the most appreciated laboratory service by respondents concerned the reliability of test results; similar findings have been reported in the USA, Yemen, and Saudi Arabia [3,20,21]. The adequacy of result reports, laboratory staff availability, and leadership responsiveness were rated with satisfaction and concurred with similar studies [17–19]. It is encouraging that these laboratory services indicators were appreciated by the physicians. Such appreciation could lay a good foundation of collaboration between laboratories and clinicians to improve identified gaps. At the bottom of the scale in 17 studied laboratory service categories were routine tests TAT followed by in-patient stat tests TAT. These categories were followed by communication of changes in laboratories. Such low ratings in these services have also been reported in Ethiopia, the USA, and Saudi Arabia [3,6,17,21]. The physicians’ poor rating of TAT corroborates the findings of assessment done across these laboratories in 2017, which found that TAT was not regularly monitored in three out of the four laboratories [15]. Even though the targets of TAT are set, sustainable improvement would only be possible if there is regular monitoring to continuously identify and address the gaps.

Communication is key for customer satisfaction. The findings of this study showed that test TAT was not communicated to the majority of physicians (84%). It is important for clinicians to know when laboratory test results are expected so that they can well manage patient communication and appointments. Not knowing tests TAT and changes to laboratories, such as interruptions and new tests or delays in reporting results, will affect clinical activities and patients’ health care. Although the expectations of physicians may differ from actual laboratory realities, especially regarding TAT, a good communication system could provide common ground and improve service delivery for both clinical and laboratory services. Additionally, the clinical services should not only wait for a one-way solution from laboratories, especially when they belong to the same institution. Reciprocal communication and a discussion platform for stakeholders could offer a better solution for service improvement. Studies recently conducted in these laboratories also found a communication gap between laboratories and clinicians and poor performance in conducting management reviews [9,11,22]. These reviews should discuss the underlined issues, strategies, and the role of each stakeholder in a laboratory improvement program. The fact that management reviews were not regularly organized may explain the gaps found, including communication.

Despite the level of the physicians’ satisfaction with overall laboratory services in general, it is also important to note that pediatricians and internists were the least satisfied compared to other specialties. One explanation for this could be that their departments use more laboratory services compared to other departments. Pediatricians in Rwanda deal more with acute diseases which require more attention and urgent responsiveness service. Urgent responsiveness is expected from the laboratory or any other supporting services vis-à-vis the attendant clinician, and when these expectations are not met, it could cause dissatisfaction. That may be a possible explanation for why these specialties are less satisfied. The more experienced doctors (>10 years of experience) were also less satisfied compared to the reference group (<1 year of experience). The same observation was made in southern Ethiopia where specialists were less satisfied with tests TAT [17]. This dissatisfaction is more likely to be explained by the critical analysis of experienced physicians versus younger ones. The variability in physicians’ satisfaction observed between hospitals could be explained by a relative difference in quality performance in these laboratories throughout different assessments [15].

In the context of this study, physicians were the most appropriate laboratory customers whose perspectives could highlight the functional state of laboratories’ quality of services, and it was limited at four referral hospitals. Future studies may focus on other laboratory stakeholders as well as at other levels of health care in the country. Nevertheless, this study was the first laboratory customer survey in Rwanda and may contribute to a laboratory quality improvement program. The high level of participation and the general open-ended question constituted the strengths of the survey. Despite the other processes of continuous laboratory improvement, which are always recommended, the physicians’ satisfaction survey was a useful analysis of laboratory services improvement. Such a survey would regularly capture the primary customers’ observations for laboratory quality service. These results could then be discussed at the institutional quality improvement fora [3,5]. The standardization and institutionalization of such surveys in the process of laboratory quality improvement would most probably have a positive impact for improvement of the quality in health care.

In conclusion, this study highlighted a number of areas for improvement to be addressed. This study offered an opportunity to mirror the country’s laboratory quality system that serves as a planning basis for laboratory services and quality healthcare improvement. Additionally, for the quality service sustainability, collaboration between clinicians and clinical laboratories should be reinforced. To that extent, laboratories cannot afford to not satisfy physicians as their primary customers. In light of this study, ensuring continuous availability of laboratory tests, timely reporting results, and effective communication between laboratories and clinicians would raise physicians’ satisfaction and most probably also result in patient benefits. However, further studies are needed to demonstrate the effect on patients. One could suggest that the scheduled participation of laboratory staff in clinical meetings and ward rounds with physicians may be a way of addressing many of the physicians’ concerns and would benefit the quality of health care.

## Paper context

Physicians’ satisfaction with laboratory services indicates key areas of improvement in laboratory services to improve the quality of health care Few studies conducted on physicians’ satisfaction with clinical laboratories lack participants’ perspectives and are not enough diversified in terms of contexts. Additional to the level of physicians’ satisfaction for studied laboratory services, this study highlights the areas of improvement in laboratory services in Rwanda according to physicians’ perspectives and their laboratory service satisfaction level.

## Appendix A. List of tests in referral hospitals

Test menu for the laboratory in hospital 1 (This list is similar to other three laboratories as they are in the same category and we presented one list to avoid too long list).

**Table:**

Test NAME	Description	TAT in Hours
A/G Ratio	Albumin/Globulin ratio	2
AFB CULTURE (BK/BAAR)	Liquide culture for TB	1008
AFB Staining (BK/BAAR)	Auramine/ziel	12
AFP	Alfa-Feto-Protein	2
ALAT/SGPT A	Alanine Amino Transferase ARCHITEC	2
ALAT/SGPT C	Alanine Amino Transferase Cobas c311	2
Albumin A	ARCHITECT	2
Albumin Body fluid	Albumin in (CSF, Ascitis, pleural, pericardial, synovial)	2
Albumin c	Cobas c311	2
Alkaline phosphatase A	ARCHITECH	2
Alkaline phosphatase C	Cobas c311	2
Alpha-fetoprotein(AFP)	RACHITECT	2
Amylase A	ARCHITECT	2
Amylase C	Cobas c 311	2
AMYLASURIE	AMYLASE IN URINE	2
APTT	Activated Partial Prothromboplastin Time	2
ASAT/SGOT A	Aspartate Amino Transferase ARCHITECT	2
ASAT/SGOT C	Aspartate Amino Transferase on c311	2
GGT A	Gamma GlytamylTransferase ARCHITEC	2
GGT C	Gamma GlytamylTransferase Cobas c311	2
Ascitis bacteriology	Cyto-Bacteriological Exam of ascitis	72
Ascitis chemistry		2
ASLO	Anti Strepto Lysine O	2
BAL bacteriology	Cyto-Bacteriological exam of bronchoalveolar lavage	72
Base excess		2
Bile salts		2
Bilirubinuria		1
FERRITIN	FERRITIN	2
HCO3-	Bicabonate	2
Calcium A	ARCHITEC	2
Calcium C	COBAS C311	2
Caliciuria	Calcium in urine	2
Caryotype	Karyotype	720
Cervical swab bacteriology	Cyto-Bacteriological exam of cervical swab	72
Chloride A	ARCHITEC	2
Chloride Body fluid	Chloride in (CSF, Ascitis, pleural, pericardial, synovial)	2
Chloride C	Cobas c311	2
Cholesterol Esters		2
CK-MB A	Creatinine Kinase Muscle Brain ARCHITEC	2
CK-MB C	Creatinine Kinase Muscle Brain Cobas c311	2
CK-MM	Creatinine Kinase Muscle Muscle	2
CO2 Tot	Carbon monoxyde total	2
Coproculture		72
CPK	CreatininPhospho kinase	2
Creatinine A	Creatinine ARCHITEC	2
Creatinine C	Creatinin Cobas c311	2
Creatininuria	Creatinine in urine	2
CRP abort	C Reactive Protein	2
CRP humatex	C-reactive protein	2
Cryptococcal Ag	Cryptococcal Antigen	2
CSF bacteriology	Cyto-Bacteriological Exam of CSF	72
CSF chemistry	Glucose, protein, LDH, Pandy	2
HDL Cholesterol A	HDL Cholesterol ARCHITEC	2
HDL Cholesterol C	HDL Cholesterol Cobas c311	2
Direct bilirubin C	Direct bilirubin Cobas c 311	2
Direct bilirubin C	Direct bilirubin ARCHITEC	2
Direct Coombs		2
DSE/EDS	Direct Stool Examination wet preparation	2
DSE/EDS Concentration	Direct Stool Examination with concetration	2
DSE/EDS Staining	Direct Stool Examination with special staining	2
EBV Ab ELISA	Antibody Anti Epstein-Barr Virus ELISA	2
EBV Ab Rapid	Antibody Anti Epstein-Barr Virus Rapid test	2
EID (DBS)	Qualitative Test	336
FBC/NFS		2
ESR/VS	Erythrocyte Sedimentation Rate	2
Blood group/Groupe sanguin		2
BT/TS	Bleeding Time	2
CT/TC	Clotting Time	2
Ascitic cytology	Cytology of ascitic fluid	2
INR	International Normalized Ratio	2
CSF Cytology	Cytology of Cerebro Spinal Fluid	2
Hemostasis		2
FIB	Fibrinogen	2
Reticulocytes		2
Sickle Cell Test		2
Sickle cell test/Test d`Emmel		7
PT/TP	Prothrombine Time	2
F PSA	Prostatic Specific Antigen	2
Estradiol	Oestradiol hormone	2
FNA	Cytopathology	2
Free βHCG	Beta Human Chorionic Gonadothropine	2
FSH	Follicular Stimulating Hormon	2
FT3	Tri-Iodo-Thyronine hormone	2
FT4	Tetra-Iodo-Thyronine	2
Gene Xpert		4
Globulins		2
Glucose Body fluid	Glucose in (CSF, Ascitis, pleural, pericardial, synovial)	2
GLUCOSURIA C	Glucose in urine Cobas c311	1
Glycemia A	ARCHITEC	2
Glycemia C	Blood glucose Cobas c311	2
Glycosylated Hb A1C/HbGlyquee A	Glycosylated Hb A1C/HbGlyquee ARCHITEC	2
Glycosylated Hb A1C/HbGlyquee C	Glycosylated Hb A1C/HbGlyquee Cobas c311	2
H-Pylori Ab ELISA	Antibody anti H-Pylori ELISA	2
H-Pylori Ab Rapid	Antibody anti H-Pylori Rapid test	2
HB Viral load	Quantitative test	720
HBcAb ELISA	Antibody anti Hepatitis B core antigen ELISA	2
HBcAb Rapid	Antibody anti Hepatitis B core antigen Rapid test	2
HBeAb ELISA	Antibody anti Hepatitis B e-antigen ELISA	2
HBeAb Rapid	Antibody anti Hepatitis B e-antigen Rapid test	2
HBeAg ELISA	ELISA test for Hepatitis B e antigen	2
HBs Ab ELISA	Antibody anti Hepatitis B surface antigen ELISA	2
HBs Ab Rapid	Antibody anti Hepatitis B surface antigen Rapid test	2
HBsAg ELISA	ELISA test for Hepatitisa B surface antigen	2
HBsAg rapid	Hepatitis B surface antigen	2
HC Viral Load	Quantitative test	720
HCV Ab ELISA	Antibody anti Hepatitis C virus ELISA	2
HCV Ab Rapid	Antibody anti Hepatitis C virus	2
Hemoculture	Blood culture	168
Histopathology/Examend’une piece operatoire		168
HIV ELISA	HIV	168
HIV Rapid	HIV	1
HIV Viral Load	quantitative Test	168
Indirect bilirubin		2
Indirect Coombs		2
IRON A	SERUM IRON ARCHITEC	2
IRON C	Serum iron Cobas c311	2
Kaliuria	Potassium in urine	2
Keton bodies	Test is performed in urine	1
LDH A	Lactate Deshydrogenase ARCHITEC	2
LDH Body fluid	LDH in (CSF, Ascitis, pleural, pericardial, synovial)	2
LDH C	Lactate Deshydrogenase Cobas c311	2
LDL Cholesterol A	LDL Cholesterol ARCHITEC	2
LDL Cholesterol C	LDL Cholesterol Cobas c311	2
LH	Luteinizing Hormon	2
Line probe Assay (Hain test)		48
Lipase A	Lipase ARCHITEC	2
Lipase C	Lipase Cobas c311	2
Lithium		2
Magnesium A	Magnesium ARCHITEC	2
Magnesium C	Magnesium Cobas c311	2
Myelogram	Bone marrow cell count	4
Nasal swab bacteriology	Cyto-Bacteriological exam of nasal swab	72
Natriuria	Sodium in urine	2
One micturation quantitative proteinuria		2
Ordinary culture		72
PBF	Peripheral blood film	2
pCO2	Partial pressure of Carbon dioxyde	2
Pericardial bacteriology	Cyto-Bacteriological Exam of pericardial fluid	72
Pericardial chemistry		2
Pericardial cytology	Cytology of pericardial fluid	2
pH Blood		0.33
pH Urine		1
Phospholipid		2
Phosphoriuria	Phosphorous in urine	2
Phosphorous A	Phosphorous ARCHITECT	2
Pleural bacteriology	Cyto-Bacteriological Exam of pleural fluid	72
Pleural chemistry		2
Pleural cytology	Cytology of pleural fluid	2
pO2	Partial pressure of Oxygen	2
Potassium A	Potassium ARCHITEC	2
Potassium C	Potassium Cobas c311	2
Pregnancy test		2
Progesterone	Progesterone hormone	2
Prolactin	Prolactin hormone	2
Prostatic acid phosphatase		2
Protein Body fluid	Protein in (CSF, Ascitis, pleural, pericardial, synovial)	2
PROTEIN IN CSF		1
Prothrombine Rate	Prothrombine Rate	2
Pus swab bacteriology	Cyto-Bacteriological exam of pus wab	72
Qualitative Glucosuria		1
Qualitative Proteinuria A	ARCHITEC	1
Qualitative Proteinuria C	Qualitative Protein in urine Cobas c311	1
Quantitative proteinuria 24 h A	ARCHITEC	2
Quantitative proteinuria 24 h C	Cobas c311	2
QulitativeGlucosuria A	Glucosuria ARCHITEC	1
Rectal swab bacteriology	Cyto-Bacteriological exam of rectal swab	72
Rhumatoid factor		2
RPR	Rapid Plasma Reagin	2
Rubella Ab ELISA	Antibody anti Rubela ELISA	2
Rubella Ab Rapid	Antibody anti Rubella rapid test	2
Semen bacteriology/Spermoculture	Cyto-Bacteriological Exam of semen	72
Skin swab bacteriology	Cyto-Bacteriological exam of skin swab	72
Sodium A	Sodium ARCHITEC	2
Sodium C	Sodium Cobas c311	2
SODIUM IN URINE	SODIUM IN URINE	2
Spermogram		48
Sputum bacteriology	Cyto-Bacteriological exam of sputum	72
St HCO3-	Saturated bicarbonate	2
Synovial bacteriology	Cyto-Bacteriological Exam of Synovial Fluid	72
Synovial chemistry		2
Synovial cytology	Cytology of synovial fluid	2
T PSA	Prostatic Specific Antigen	2
T T3	Tri-iodo-thyronine hormone	2
Testosterone	Testosterone hormone	2
Thick smear+Parasitemia	GE + parasitemia	1
Throat Swab bacteriology	Cyto-Bacteriological exam of throat Swab	72
TIBC A	Total Ion Capacity Binding ARCHITEC	2
TIBC C	Total Ion Capacity Binding Cobas c311	2
Total acid phosphatase		2
Total bilirubin A	Total bilirubin ARCHITEC	2
Total bilirubin C	Total bilirubin Cobas c311	2
Total cholesterol A	Total cholesterol ARCHITEC	2
Total cholesterol C	Total cholesterol Cobas c311	2
Total glycosylated Hb/HbGlyquee		2
Total lipid		2
Total protein A	Total protein ARCHITEC	2
Total protein C	Total protein Cobas c311	2
TOTAL βHCG	Beta Human Chorionic Gonadothropine	2
Toxoplasma Ab ELISA	Antibody anti Toxoplasma ELISA	2
Toxoplasma Ab Rapid	Antibody anti Toxoplasma Rapid test	2
TPHA	TreponemaPallidumHemaglutination Essay	2
Transferrin		2
Triglycerides A	Triglycerides ARCHITEC	2
Triglycerides C	Triglycerides Cobas c311	2
Troponin I		2
TSH	Thyroid Stimulating Hormone	2
TT	Thrombine Time	2
TT4	Total Tetra-iodo-Thyronine	2
Urates blood		2
Urates urine		2
UREA A	UREA ARCHITEC	2
UREA C	UREA Cobas c311	2
Ureauria	Urea in urine	2
Urethral swab bacteriology	Cyto-Bacteriological exam of urethral swab	72
URIC ACID A	URIC ACID ARCHITEC	2
URIC ACID C	URIC ACID Cobas c311	2
Urinalysis/ECBU	Cyto-Bacteriological Exam of urine	72
Urine amylase		2
Urine density		1
Urobilinogenuria		1
Urobilinuria		2
Vaginal swab bacteriology	Cyto-Bacteriological exam of sputum vaginal swab	72
VDRL/RPR	Manual	2
Vitamin A	Automated	2
Vitamin B12	Automated	2
Wet preparation		1
